# Chronic idiopathic musculoskeletal pain in youth: a qualitative study

**DOI:** 10.1186/s12969-019-0389-3

**Published:** 2019-12-27

**Authors:** Hervé Lefèvre, Alexandra Loisel, Brigitte Bader Meunier, Chantal Deslandre, Noémie Lemoine, Marie Rose Moro, Pierre Quartier, Jonathan Lachal

**Affiliations:** 1 AP-HP, Cochin Hospital, Maison de Solenn, Paris, France; 2 CESP, Fac. de médecine - Univ. Paris-Sud, Fac. de médecine - UVSQ, INSERM, Université Paris-Saclay, 94805 Villejuif, France; 3 Université de Paris, PCPP, Boulogne Billancourt, France; 4 French Clinical Research Group in Adolescent Medicine and Health, Toulouse, France; 5 APHP, Trousseau Hospital, Paris, France; 6 Université de Paris, Institut IMAGINE, Centre de Référence National Pour les Rhumatismes Inflammatoires et les Maladies Auto-Immunes Systémiques Rares de l’Enfant (RAISE), Paris, France; 7 APHP, Necker-Enfants Malades Hospital, Unité d’Immunologie, Hématologie et Rhumatologie Pédiatrique, Paris, France; 8 Unité d’Immunologie, Hématologie et Rhumatologie Pediatrique, Hôpital Necker-Enfants Malades, Assistance Publique Hôpitaux de Paris, 149 Rue de Sevres, 75015 Paris, France

**Keywords:** Adolescent, Chronic pain, Qualitative research, Musculoskeletal

## Abstract

**Background:**

Chronic musculoskeletal pain (MSP) is frequent in adolescents and has major medical and social consequences. In many cases, when no cause has been clearly established, this pain may be considered to be chronic idiopathic MSP. Our study seeks to identify general criteria for this type of pain through the experience of professionals from tertiary care centers with expertise in pediatric and adolescent chronic MSP.

**Methods:**

Cross-sectional multicenter qualitative study. Semi-structured interviews of 25 professionals at a rheumatology reference center and in its network for pain management, including diverse specialists and professions. Interpretative Phenomenological Analysis is used to explore the data.

**Results:**

This approach led us to identify 10 themes organized around three superordinate themes covering different stages of the diagnostic process: 1) the medical pain history up to the consultation at the reference center; 2) the professional’s subjective feelings about the clinical presentation; 3) from the clinical examination to diagnosis and treatment of chronic idiopathic MSP. The main elements guiding this diagnosis do not come from the physical examination but from the medical history and the professionals’ subjective feelings, that is, their clinical judgment. The professionals’ impression of uneasiness and frustration, induced by patients and their parents, is of major importance.

**Conclusion:**

The principal elements guiding the diagnosis of chronic idiopathic MSP do not come primarily from the physical examination but rather from the pain history and the health professional’s subjective feelings. Our results suggest that the concept of Juvenile Fibromyalgia (JFM) does not appear to cover all situations of chronic idiopathic MSP in adolescence. A constellation of non-organic criteria enables diagnosis of the latter; these criteria should be validated to avoid medical nomadism and multiple investigations and to shorten the interval until patients receive optimal pain management.

**Clinical trial registration:**

clinicaltrials.gov, NCT03171792, https://clinicaltrials.gov/ct2/show/NCT03171792?term=LACHAL&cntry=FR&city=paris&rank=1

## Background

Musculoskeletal pain (MSP) is one of the principal types of pain in adolescents [1–4]. Chronic MSP is defined as pain of the joints and soft tissue that lasts longer than 3 months and affects the patient’s daily activities. It is frequent [5], with a prevalence ranging from 7 to 15% between studies and increasing with age and puberty; it is more prevalent among girls [6, 7]. The prevalence of MSP is high, appears to be increasing, and may affect primary care provision [8] and quality of life [3, 9–11].

Causes of Chronic MSP and the duration of its development determine where and by whom it is managed. Three quarters of the MSP cases seen by physicians in private practice result from injuries, mechanical/overuse syndromes, and osteochondrosis [3, 12]. In some pediatric rheumatology centers, juvenile fibromyalgia (JFM), complex regional pain syndrome, local pain syndrome, or low back pain account for 25% of their diagnoses [13] and half of new patients there are referred for chronic MSP [14]. These specialized departments diagnose and manage inflammatory diseases affecting the joints or musculoskeletal system [15] and also offer consultations for a second opinion on the diagnosis of complex cases of such pain. This assessment is often based on a set of clinical, paraclinical, and therapeutic features. Some diagnostic causes are based on converging lines of evidence or diagnostic scores [3, 10, 16, 17]. Chronic MSP in adolescents can be due to a variety of etiologies including traumatic, developmental, and inflammatory (most frequently, juvenile idiopathic arthritis). The various types of chronic MSP of an undetermined cause are typically classified as chronic idiopathic MSP.

For the past two decades, researchers have worked hard to improve our understanding of this entity and its management. As part of this work, studies about the diagnostic criteria for fibromyalgia have enabled this management to advance [18–23], under the impetus of the American College of Rheumatology. These criteria were recently updated for JFM [16].

JFM is considered to be complex chronic pain, apparently part of a larger group of diffuse chronic pain syndromes among children and adolescents. Its diagnosis can be difficult in view of the concomitant presence of symptoms including cognitive, sleep, and mood disorders, other pain symptoms (headaches and abdominal pain), joint hypermobility, and dysautonomia [18]. A JFM diagnosis does not explain all cases of chronic idiopathic MSP among children and adolescents. Moreover, there is no unanimous consensus today either about this chronic idiopathic diagnosis or about its management [24, 25].

In these situations, a diagnosis in primary health care can be difficult, especially when physical and psychological symptoms coexist, when doctors recurrently worry about missing the diagnosis of an organic disease [9] or do not find enough specific criteria.

Although the physiopathology of chronic pain is better understood today, many consider a diagnosis of chronic idiopathic MSP to be a diagnosis of exclusion, while others treat it as JFM with the limitations of its diagnostic criteria. In this context, the existence of global positive specific criteria for chronic idiopathic MSP remains to be demonstrated [26, 27]. Our study explores the experience of healthcare professionals who are experts in chronic MSP in adolescents, to analyze their clinical experience with these teens and their families and to investigate the criteria they use to move forward in their diagnoses and the factors supporting a diagnosis of chronic idiopathic MSP.

## Method

This multicenter, cross-sectional, observational, non-interventional study followed a qualitative, phenomenological, and inductive process.

We aimed to interview healthcare professionals working at the 3 sites of the Rare Auto-immune and Inflammatory Systemic diseases (RAISE) pediatric rheumatology reference center, including one department of pediatric rheumatology and a pain center at Necker Children’s Hospital, one general pediatric department and a pain center at Robert Debré Hospital, and an adult rheumatology department that provides consultation and follow-up for older adolescents and young adults at Cochin Hospital. All three hospitals are part of the Paris public hospital system (AP-HP). Sampling was purposive, with maximum variation to obtain the widest possible diversity for this theme. The participants could be physicians or allied health professionals (Table 1). Experience in the management of patients with chronic MSP was the principal criterion for recruitment.

**Table 1:** Participants’ Characteristics

ID	Profession	Workplace	Age of patients (years)	Age group professionals (years)	Years of practice
Participant 1	Pediatrician	Internal medicine dept.	< 18	30–40	3
Participant 2	Pediatrician	Internal medicine dept.	< 18	50–60	22
Participant 3	Orthopedist	Pain center	< 18	40–50	15
Participant 4	Pediatrician	Rheumatology	< 18	50–60	30
Participant 5	Anesthetist	Pain center	< 18	50–60	30
Participant 6	Rheumatologist	Rheumatology	> 15	60–70	30
Participant 7	Rheumatologist	Rheumatology	> 15	40–50	20
Participant 8	Osteopath	Pain center	< 18	30–40	9
Participant 9	Pediatrician	Rheumatology	< 18	50–60	25
Participant 10	Psychologist	Pain center	< 18	30–40	8
Participant 11	Rehabilitation physician	Rehabilitation center	< 18	50–60	30
Participant 12	Pediatrician	Internal medicine dept.	< 18	30–40	10
Participant 13	Rheumatologist	Rheumatology	> 15	40–50	13
Participant 14	Physical therapist	Internal medicine dept.	< 18	30–40	15
Participant 15	Psychologist	Pain center	< 18	30–40	11
Participant 16	Physical therapist	Pain center	< 18	50–60	23
Participant 17	Pediatrician	Rheumatology	< 18	50–60	22
Participant 18	Pediatrician	Rheumatology	< 18	50–60	20
Participant 19	Pediatrician	Pain center	< 18	30–40	10
Participant 20	Psychologist	Internal medicine dept.	< 18	50–60	30
Participant 21	Pediatrician	Internal medicine dept.	< 18	40–50	25
Participant 22	Physical therapist	Rheumatology	< 18	30–40	18
Participant 23	Osteopath	Pain center	< 18	30–40	6
Participant 24	Occupational therapist	Rheumatology	< 18	50–60	27
Participant 25	Psychiatrist	Pain center	< 18	30–40	10

Data were collected during audio-recorded semi-structured individual interviews, each lasting around one hour (Table 2). The audio recordings were then transcribed and anonymized.

We used Interpretative Phenomenological Analysis, an established qualitative methodology used to explore in depth how individuals perceive particular situations they are facing and how they make sense of their personal and social world [13, 17, 26]. The approach is phenomenological, in that it involves a detailed exploration of participants’ experiences. The aim is to explore their personal experience and subjective perception of an object or event. Rather than reducing a phenomenon to a number or an identifiable variable and controlling the setting in which it is studied, phenomenology aims to describe it as faithfully as possible in the setting in which it occurs.

Each theme is related to specific statements from the transcript. The analysis was performed independently by four researchers (HL, NL, AL, and JL), with NVIVO11® software, and our results were sent to participants for comment. This report meets the COREQ criteria.

## Results

For this study, we interviewed 25 professionals (Table 1); all those we contacted agreed to participate. We organized our results around three superordinate themes (Tables 3 and Additional file 1**:** Table S4 contain the direct quotations**)**.

**Table 2:** Interview Guide

1) How could you define chronic idiopathic musculoskeletal pain? 2) Could you explain to me what you consider to be the main elements that help to differentiate it from other organic or mixed conditions? 3) What is your clinical approach to a clinical picture suggesting chronic idiopathic musculoskeletal pain?

**Table 3:** Verbatim quotations from participants

Narrative of the healthcare pathway	
A long medical pathway	Diagnostic delay: [They’ve had a] medical pathway that is very long, they’ve seen 12 million doctors with tons of medical hypotheses (P10). Medical nomadism: A past, years, years of pain, several MRIs, scintigraphies, examinations of everything, no one’s found anything (P7). Long course: A patient told us, I’ve been in pain for 15 years […]. If no diagnosis has been reached in 5, 10, or 15 years, that already means first, it’s not severe, which is already reassuring for the patient, but also that it may not be organic (P13).
Clinical history suggestive of chronic idiopathic MSP	Precipitating factor: Often there’s a history of trauma that can sometimes be very anecdotal […]. There was a little thing that happened and as a result a whole set of things crystalized in a place in the body (P3). Pain scores: Patients who always give themselves a 10, it’s almost never organic (P16). Pain description: Very atypical pain: it burns, it stings, that’s not very organic (P4); Impalpable/intangible pain, very diffuse, at any point, is going to lead me to suspect it’s partly psychogenic (P17). Normal clinical and paraclinical examinations: If I find nothing, I go more towards something functional (P1); When there are diagnostic doubts and all the stages and examinations that were done … come back normal, that points toward functional (P6). Consequences: Functional consequences ... seem to me very important in term of disconnecting from school (P17). Family history: We’ve had families where finally in fact where everything had been constructed from generation to generation for two or three generations of spondylarthritis when in fact no one had spondylarthritis (P13).
A feeling of medical/pharmacological impotence	When there are many different treatments used and none were effective, that sets off an alarm (P12). Nothing works and/or nothing is tolerated because sometimes when nothing is tolerated, that’s a little special too (P19).
The subjective elements of the clinical presentation and the professional’s feelings	
The adolescents’ grievances	“It’s easier for the patient to hold on to something objective by saying, I have rheumatoid arthritis, so they will find a treatment that makes me feel better, whereas if there’s nothing very specific and he’s already tried 36,000 drugs, he says to himself or at least he can say to himself, how can I get better? (P13).
Reorganization of family functioning	Symbolic role: The pain becomes a member of the family in its own right (P19). Dysfunctional reorganization: If we take away the child’s pain, there’s no more family system, they don’t know how to function (P20). Parental reactions: They are always disappointed, they are always disappointed that we haven’t found an organic disease. In the parents’ mind, organic means that we can treat it (P4).
The professionals’ feelings	Difficulty, doubt, ignorance: Over time, I realize that we are all so hopeless, so embarrassing, and that there are no very objective criteria (P4); I am never very comfortable: the diagnosis of functional pain hides a great deal of ignorance (P4). Impotence, frustration Some doctors feel frustrated, helpless. It can go as far as conflict... What frustrates me deeply is just to not be able to help them (P15); We feel helpless because we cannot relieve their pain (P19). Long consultations These are consultations […] that are extremely time-consuming, require a lot of attention, and it’s exhausting (P12).
The function of pain	The symptom is at two levels, it is first individual, for the subject, it has a place, a function, for the patient, and after that, it becomes part of a family system (P10); When physicians raise the question of the function of the pain, it is perhaps necessary to refer the patient pretty rapidly towards a pain consultation (P19).
From the clinical examination to a diagnostic and treatment synthesis of chronic idiopathic MSP	
Clinical examination	Absence of relevant objective diagnostic elements: A knee that is totally flexible, a girl who is on her legs, standing, without pain, it’s certainly not inflammatory joint pain (P4). Existence of sleep disorders without nocturnal pain: They always talk about [pain-free] fatigue even though their nights are all right (P7). Constellation of associated functional symptoms: Permanent fatigue, headaches, symptoms that absolutely do not correlate with what the paraclinical results or the treatments given (P17).
The elements of psychological symptomatology	Relational difficulties with peers: They are not very socially skilled at approaching others (P25). School absences: The less organic it is, the more school absences there are (P6). Performance anxiety: We often find often […] really substantial anxiety in relation to performance, whether it’s sports or school (P18). A predominant psychological dimension: I am going to feel stronger resistance when there is a strong psychogenic proportion than when it’s a purely organic disease (P17).
The diagnostic procedure and treatment tests	Clinical monitoring: The question of the order in time is very important (P11). Difficulties in using the diagnosis of fibromyalgia: The most difficult situation, it’s the pain that’s enthesopathic, that’s really the hardest because objectively, a purely organic enthesitis can given exactly the same pain on first view, as, in quotation marks, fibromyalgia, a term that I never use, by the way, for fear of letting patients latch onto the idea that they have a chronic disease and fall into the associations of fibromyalgia patients and all that (P3). Test treatment: When I do not manage to have enough information to decide if it’s functional or organic, I propose a treatment for 2 weeks by NSAID, stop for 3 days, and 2 weeks of step 2 analgesics. What I want to know is the answer to the question: which of these 2 treatments helped most? (P6).

### Narrative of the pain story

#### A long medical peregrination

As stated above, chronic idiopathic MSP is a diagnosis of exclusion: *it can be diagnosed only when there is no alternative explanation of the symptoms*. The multiplicity of clinicians seen throughout the length of the medical follow-up is the first evidence of diagnostic delay. It may be related to the difficulty of ruling out an organic cause for the pain or of treating it (for physicians) or of hearing the diagnosis (for adolescents and their families). The large number of different specialists is a further source of confusion and makes the parents’ requests still more complex.

This medical nomadism (a form of doctor-shopping, but for a diagnosis or second or third opinion rather than the form intended to procure medication) is associated with the repetition of various examinations, all or most with non-specific results; it is highly suggestive of chronic idiopathic MSP. The duration of the pain and the substantial delay between its onset and the consultation are paradoxically reassuring factors for the physician regarding the absence of an underlying severe organic disease. Nonetheless, it sometimes happens that an erroneous diagnosis of this chronic idiopathic disease is corrected in favor of a more specific cause.

#### A clinical history suggestive of chronic idiopathic MSP

The pain history is fundamental. The professional asks first about a precipitating factor, in cases not associated with inflammatory pain. Physical or psychological trauma, often long ago, is frequent. It was often benign and not a source of concern at the time. Depending on the context, the pain may be likely either to become chronic or to be reactivated in the presence of new trauma.

The description of the pain is a useful diagnostic element. Often put in the foreground, its description is nonetheless often vague. It is constant or develops in random flares, increasingly intense and unbearable. It is difficult to score on a scale, and its high score does not change.

The repeated clinical examinations must be interpreted as normal, with no clinical anomaly observed beyond the potential consequences associated with the pain experienced, sometimes with some hypermobility and/or prolonged immobilization.

The consequences are often important and long-lasting: absenteeism, disconnection from school, school failure, harassment, relationship difficulties, withdrawal into self, and isolation.

Family history is often a confounding factor: diagnostic errors of organic causes of pain that may be intergenerational. In some families, pain develops from diseases that may be constructed, self-diagnosed, or undiagnosed chronic pain syndromes.

#### A feeling of medical/pharmacological powerlessness

Patients describe the different medical treatments as ineffective. Extreme but transient effectiveness, called *magical*, is sometimes reported, but its rapid exhaustion suggests a placebo effect. Powerful analgesic treatments are often poorly tolerated. Medical inadequacy in treating the patient’s pain, by disappointing the family, can reinforce medical nomadism and sometimes lead to dangerous treatment escalation.

### Subjective elements of the clinical presentation and professionals’ feelings

#### The adolescents’ grievances

Patients with chronic idiopathic MSP express strong **grievances**. The absence of an organic diagnosis and of effective treatment leave the family dissatisfied with the physician; the patients feel misunderstood. The social consequences of the pain, most importantly, the disconnection from school, validate its intensity. They attribute their imprisonment in their pain to the inability of doctors or the field of medicine, more generally, to understand their situation.

#### Reorganization of family functioning

This chronic idiopathic pain often takes on an important symbolic role in the family organization. The parents may overprotect the child, who withdraws sometimes to let them explain the situation. Pain can play the role of organizing, managing the family’s functioning. It sometimes seems to be maintained, ostentatiously perpetuated – by a wheelchair – despite the absence of objective evidence, and it is used inappropriately to justify the time spent away from school. Dysfunctional family reorganization, secondary benefits to a disease, or inappropriate investments in patient associations are suggestive of chronic idiopathic pain.

Parental anxiety promotes medical nomadism. Paraclinical examinations (tests, imaging) intended to reassure the family instead maintain doubt and are experienced by the family as the reiteration of diagnostic errors. The absence of mutual understanding between the family and the doctor can lead to conflict.

#### The professionals’ subjective feelings

The doctor’s feelings, which are essentially implicit or subjective clinical judgments, are a supplementary indication that can guide the diagnosis. They frequently describe feelings of embarrassment, helplessness, and frustration.

Physicians often feel uncomfortable in broaching the unexplained nature of the pain. They experience difficulty in finding its origin, and the often hostile reaction of the adolescents and parents reinforces their discomfort. They may feel that their clinical expertise is not valued.

The discomfort is enhanced by their feeling that they are failing to meet the family’s expectations. Their reassuring discourse is misperceived.

Consultations for chronic idiopathic MSP are complex, time-consuming, and difficult, as the professionals search for something to orient their diagnosis. The absence of elements of diagnostic certainty can encourage the physician’s persistent worry.

#### The function of pain

Wondering about the role or function of chronic pain essentially responds to a sense that it is functional, that is, not organic. Organic pain has no function other than to tell patients that something is wrong and needs addressing as an organic process, while chronic idiopathic MSP can have several functions. From a way of expressing personal or family problems, difficulties of family cohesion, or as an alarm signal for a situation the adolescent is finding it difficult to live through, it can sometimes provide a protective identity for an adolescent experiencing insecurity or vulnerability in the face of the social or school system.

### From the clinical examination to diagnosis and treatment of chronic idiopathic MSP

#### Clinical examination

A meticulous clinical examination finds no relevant objective diagnostic factors and no growth abnormalities affecting weight or height. The fatigue is often associated with sleep disorders that do not involve nocturnal pain. The presence of a constellation of functional-type signs orients the doctor towards a diagnosis of chronic idiopathic MSP: tension headaches, non-specific abdominal pain, or ill-defined pain in multiple locations.

#### The elements of psychological symptomatology

Adolescents with chronic idiopathic MSP are described as fairly intelligent, but often seen to have troubles with their peers and to get along more easily with adults.

Social inhibition, performance anxiety, and to a lesser extent, signs of depression are frequent. The anxiety has sometimes been aggravated by diagnostic delay and the fear of serious disease. Catastrophizing is thus present; the goal is to pinpoint its origin.

The suggestion of a psychological cause may be refuted quite assertively, or, inversely, very smoothly with the denial of any problem.

The set of consequences of this chronic idiopathic pain and their social intensity tend to point to a more predominant psychological dimension.

#### The diagnostic procedure and treatment tests

Once this evidence is collected, the monitoring phase begins; both clinical and paraclinical, it aims to judge the course of the symptoms and their origin, and to discuss a test treatment.

At this stage, sometimes in the presence of isolated enthesitis or diffuse pain, the pediatric specialists we questioned say they do not diagnose JFM, which they do not know well and the origin of which they do not completely understand. Most often, they avoid this diagnosis to prevent the risk that, through the patient’s and family’s organization around it, it will become chronic.

The treatment response to a test of analgesic and/or anti-inflammatory treatment, depending on the response, provides supplementary evidence of the diagnosis.

## Discussion

Our qualitative exploratory study is the first to question a group of specialists in chronic MSP about their diagnostic process for a potential diagnosis of chronic idiopathic MSP and their feelings regarding the causes and consequences of the pain in their patients and the patients’ families.

The qualitative method is particularly appropriate for the complexity of our topic. It makes it possible to describe in greater detail and depth the issues around the evaluation of this chronic idiopathic pain and thus to understand it better. Although our results are subjective and only represent the stances of our participants, a certain degree of theoretical generalization can be reached.

The analysis of our interviews with these professionals expert in chronic MSP allows us to describe the signs they use for a positive diagnosis of its idiopathic form. These items, which can be grouped together and tested as its positive diagnostic criteria, are, first of all, such explicit elements as the pain history and its context – medical nomadism at risk of diagnostic delay [17]; a history, often quite old, of trauma, and poorly defined family issues of pain [28]; meager clinical signs besides the pain in the foreground, intense, vague, often difficult to assess or challenged in the past [29, 30]; or severe school-related or social repercussions [2]. But professionals must also include implicit, more subjective factors. These concern first their perception of the patient and the family, whom they see as disappointed at the impotence of medicine to help them and whose anxiety can sometimes reorganize the family’s functioning around the symptom, to the point of making it increasingly inaccessible to any management [29, 30]. They also involve the professionals themselves, who must paradoxically use as positive clinical evidence of chronic idiopathic MSP their own frustration at the inefficacy of pharmacological treatment, their apprehension of the family’s reactions in this setting [29], and the difficulty of convincing the adolescent and the family that their pain is idiopathic, while remaining vigilant about any evolution suggesting an organic cause [2]. These feelings, inaccessible on clinical and other examinations, are nonetheless regularly expressed by the professional and considered to support this diagnosis.

The stages to pinpoint the origin of chronic idiopathic MSP and prescribe an effective treatment for adolescents with chronic pain are frequently too long and stigmatizing [31]. The invariably lengthy and complex healthcare follow-up engenders a substantial risk of physical, psychological, social, and family consequences and increases the likelihood that the pain will become chronic and treatment-resistant [17, 32]. To improve the management of adolescents, an early, validated diagnostic score for the overall category of chronic idiopathic MSP needs to be built. Its benefits might allow physicians to mention this diagnosis factually with adolescents and their families. This might in turn improve their level of confidence in the doctor, which is essential for effective management [32]. The effect might be still greater if such a score were really used by primary care physicians facing these situations, to prevent medical nomadism, the multiplication of expensive supplementary examinations, and the risk of poor interpretation, at-risk polypharmacy, and the catastrophizing of the families and sometimes the physicians themselves [28, 32, 33]. It might also limit treatment escalation and enable early orientation and referral of these patients in pain toward facilities specialized in pain management to prevent the risk that it or something similar to it will become chronic in adulthood [17, 29].

The professionals interviewed in this study, in particular those specialized in working with children and adolescents, did not use the diagnosis of JFM or its diagnostic criteria in the context of chronic idiopathic MSP. The recent changes in the JFM criteria underlie the willingness to assess the origin of diffuse chronic pain from a validated score of a scale that can be performed by a primary-care physician during a consultation, as a supplement to a careful clinical examination and additional supplementary tests and imaging if necessary [25]. Nonetheless the participants in our study do not yet consider JFM to be well-defined and are concerned that it remains at risk of becoming chronic without a specific treatment protocol. The changes in its diagnostic criteria over the years may appear suggestive of a still incomplete diagnostic process [16, 25]: i) it has not yet been validated for adolescent boys, and the stability of the diagnosis has not yet been tested by social, ethnic, or cultural variations; ii) it is insufficient because it mandates a minimum number of pain sites, which does not correspond to all chronic cases of less diffuse idiopathic MSP; iii) it does not take medical subjectivity into account, although it is a major diagnostic factor in cases of idiopathic chronic pain and its individual and family presentations and repercussions; iv) especially, there is no international consensus about the origin of JFM or the diagnosis and management of chronic widespread pain [24, 25]. The repeated modifications of the diagnostic criteria for fibromyalgia in adults, and the validation of the criteria for JFM in 2016 on the basis of the criteria validated in adults in 2010 and substantially modified since then bolster the uncertainty [16, 23].

Our results propose items other than those validated for JFM to establish a diagnosis of chronic MSP as early as possible. This study is a first step towards the construction of a global positive diagnostic score supporting an idiopathic cause of this type of chronic pain. This score will complement the existing scores for its diagnosis. It will accordingly take into account the diversity of the components of pain and facilitate access to the multidisciplinary care useful in pain management. Non-pharmacological treatment (e.g., physical and/or psychological therapy) are often more effective in this indication [34–37], as in the treatment of JFM [38–41].

Our study had some limitations. Prudence is needed about generalizing these results at the international level, principally from a cultural perspective. Pain is a symptom very sensitive to cultural components and can vary considerably from one country to another [33, 42]. It would be useful to conduct more studies in different countries to adapt these results to different cultural contexts. The accounts of patients and their families may also provide valuable assistance in improving the understanding and care of this disease [28, 31].

## Conclusions

This exploration of the experience of 25 experts in the management of chronic MSP has made it possible to describe and develop the narrative and clinical symptomatologic elements by which they implicitly orient their diagnosis toward the idiopathic form. Their experience allows us to conclude that the principal elements guiding the diagnosis do not come from the physical examination but rather from the healthcare follow-up and the health professionals’ subjective feelings and clinical sense. A constellation of non-organic criteria can help specialists to reach the diagnosis of chronic idiopathic MSP. Our study thus has potential clinical implications.

## Supplementary information

**Additional file 1:Table S4.** Other verbatim quotations from participants

## Data Availability

Table 1. Participants’ characteristics. Table 2. Interview Guides. Table 3. Verbatim quotations from participants.

## Abbreviations

FM: Fibromyalgia
JFM: Juvenile-onset fibromyalgia
MSP: Musculoskeletal pain
RAISE: Centre de compétence des rhumatismes inflammatoires des maladies auto-immunes systémiques rares de l’enfant
