# Supplementary material for: Chronic idiopathic musculoskeletal pain in youth: a qualitative study
Source: Pediatr Rheumatol Online J. 2019 Dec 27;17:86. doi: 10.1186/s12969-019-0389-3 (PMC6935211; doi:10.1186/s12969-019-0389-3)
Supplement: Supplementary file 1 — Additional file 1:Table S4. Other verbatim quotations from participants [file 12969_2019_389_MOESM1_ESM.doc]

**Table S4 – Other verbatim quotations from participants**

| **Narrative of the health care pathway** | | |
| --- | --- | --- |
| **A long medical pathway** | **Diagnostic delay** | Lots of diagnostic delay (P11)  So they were obliged to go see X who prescribed this, but it didn't work well, and X wasn't really very nice, and then after he went to see Y, yeah cortisone, it didn't work. I tried methotrexate, yes, but it upset his stomach. He went to see Z, who said, "ah, well, yes, so I'll use a biological therapy etc, etc. (P13) |
|  | **Medical nomadism** | The hardest is when they've been medical nomads. (P5)  I still ask myself questions because she had seen I don't know how many people, plenty of work-ups, all these things that were all normal. (P6)  She arrived obviously with an enormous file […]. In short, a long history. (P8)  The woman there? She's seen at least 5 physicians, 4 or 5 different rheumatologists in the last 2 years, and when I went through her test results, she had had 4 MRIs, 2 or 3 CT scans, X-rays, etc. And that's just ... suspicious. (P13)  I still remember a 15-year-old [girl] who arrived with a file like that: "Yes that's my file" and she started to present… (P5) |
|  | **Long course** | A migratory pain that's lasted three years, we're not going to worry very much. (P4) |
| **A clinical history suggestive of** **non-organic Chronic MSP** | **Pain history** | A patient who presents pain that is practically non-modifiable by any therapeutic intervention […] with no characteristic hour for inflammatory disease, and with a functional consequence that seems very important in terms of disconnecting from school. (P17)  I find that the history is essential, it can already make you understand that the picture is much more complex than it appears. (P9) |
|  | **Precipitating factor** | My key question is: have there been any recent modifications in the child's environment? (P6)  When you question them well, when you look, there was often something organic at the start, at the very beginning. (P5)  Pain doesn't fall from the sky, there was this sprain. (P10)  It's true that it started around the grandfather's death, and it was the parents who brought me this information, sort of on a platter. (P17) |
|  | **Pain scores** | [It] often seems paradoxical because it can be a teenager who marks his pain at 8/10 all the time, and nonetheless he goes to see his friends and all that. (P1)  A patient who hurts all the time, it's less organic. (P13)  The patients who refuse to score their pain, .../… that already arouses a little suspicion. (P5) |
|  | **Presentation of the pain** | An extremely painful presentation in a wheelchair with both parents [and] a procedure that looks like absolutely nothing. (P7)  If there is no description of the pain, that's a warning sign. (P19) |
|  | **Pain description** | I hurt everywhere and I hurt all the time. (P6)  If they answer "yes" to every one of my questions, I find that a little strange. (P12)  For me, the expression "It hurts everywhere" is the exact opposite of an organic disorder. (P13)  Logorrhoea that never stops, the description of all the pain, saying, my shoulder hurts, my […] hurts and even my toes; that too is rather functional. (P13)  Most often the locations change. (P21)  One of the major characteristics is that [functional] pain is continuous. (P2) |
|  | **Clinical examination** | If each time that I touch somewhere, they say "ouch", I say to myself, that's very diffuse. (P4)  Sometimes, they forget at that moment how to do it, so we have to redo all of the manoeuvres of the exam to be as objective as possible. (P6) |
|  | **Paraclinical examinations** | When I have no objective clinical sign so far. When the paraclinical examinations are negative, especially the laboratory tests… (P18)  When they arrive with a whole pack of exams and they are all negative and they ask me for new tests, that is a warning sign, in any case. (P12) |
|  | **Consequences** | Their complaint is more serious. (P5)  There are kids who for years and years are in super pain, need morphine, don't go to school, you never seen anything on the x-rays or the clinical examination… it's a little strange. (P4) |
|  | **Family history** | Chronic pain symptomatology for all the siblings with no evidence of HLA B27 spondyloarthropathy, it's nonetheless pretty suspicious. (P4)  The parents say very willingly 'and I have a pain syndrome too'. (P4)  Finally, you realize that no one ever really diagnosed it, it's a self-diagnosis. The mother says, 'I was the same at her age, she's like me, or I'm like her'. (P10) |
| **A feeling of medical/pharmacological impotence** | | They've already had NSAIDs and that did nothing. (P4)  They say, 'the treatments, they don't work'. (P5)  [The treatment] doesn't work anymore or it has no effect. (P8)  They tell you, 'it's 8, it's 9' despite the anti-inflammatory or analgesic treatment. (P18)  You can do what you want, there's not much that works. (P7)  This attitude that's 'it doesn't work at all' or 'it stops working very fast', all of them, repeatedly. (P19)  With medication, it won't work. Time is what will let things get better. (P2) |

| **The subjective elements of the clinical presentation and the professional**'**s** **feelings** | | |
| --- | --- | --- |
| **The adolescents' grievances** | | Its good news that there is no inflammatory disease, but [the patients] are always disappointed. (P4)  It's easier for the patient to hold on to something objective by saying, I have rheumatoid arthritis, so they will find a treatment that makes me feel better, whereas if there's nothing very specific and he's already tried 36,000 drugs, he says to himself or at least he can say to himself, how can I get better? (P13)  They are not happy to not have any more treatment, any more wheelchair… (P21) |
| ***Reorganisation of family functioning*** | **Symbolic role** | The symptom becomes important in the family construction. (P1)  The pain brings the parents together around the symptom.(P10) |
|  | **Dysfunctional reorganisation** | The symptoms are maintained by the parents, who are protecting the child; "That makes the whole family dysfunctional, life revolves around that." (P3)  The family environment, practically self-perpetuating. (P4)  That structures the family. (P6)  It induces family dislocations and relational problems with the sibs. (P10)  It's even harder to deconstruct what already exists. (P17) |
|  | **Parental reactions** | One of the things that warns us is if we feel that parents are ultra- anxious, feel powerless, because the pain might be a synonym for a disease that we have not discovered despite the enormous quantity of tests performed. (P19)  Chronic pain, that necessarily induces parental reactions and worry, their saying, we have to find… In any case, parents always arrive in quest of an organic cause and a treatment. (P4)  They [parents] don't like not having a diagnosis. (P7)  The parents don't accept it, they say, 'but no, I don't agree with you'. (P7) |
| ***The professionals' feelings/perceptions*** | **Difficulty, doubt, ignorance** | I am, in any case, the type to be suspicious: [I verify that] I make sure I've made a checklist of everything that's annoying. It's really the most complex kind of management, for chronic pain.  (P12)  I find it very complicated, this pain syndrome.(P4)  My experience is that it's very difficult and that you don't always succeed. (P7)  I often tell my patients that pain is something very complex …/… it's always a little grey. ((P12) |
|  | **Impotence, frustration** | You have to learn to be humble and also to not want to have a result. (P12)  I prefer to have a consultation where I feel that I understand what is going on, that my knowledge is useful to the patient, that's a good feeling. (P12)  It's difficult, sometimes you just want to say to him, 'now listen, just stop this a little, this… your carrying on'. (P16)  The physician also at one point, he's sick of it. (P21)  They are patients who are not highly regarded, because the parents are often complicated, and the children are the same. (P1) |
|  | **Discomfort** | We started with something on the order of doubt. When we don't succeed in having a certain grasp of what's going on in the body in pain, that we don't understand, that sets off things in the relationship that are extremely complicated and that can rapidly, I think, become twisted. (P20) |
|  | **Long consultations** | It's a good hour, an hour and a half consultation. (P5)  I have to give myself time when I have no results supporting an inflammatory disease. (P4)  It's not far from an hour, these appointments, it's hell…  (P6)  It's still longer because it's utterly necessary to try to find an objective element that will allow us to correct the diagnosis. (P7) |
| **The function of pain** | | Pain is as good an excuse to not be able to play football as any other. (P9)  The pain is sometimes a warning sign to seek attention or a way of expressing things that can be hard to say in words. (P15)  Protective pain [is] the hardest to demolish. (P19)  Pain [is] the instrument for expressing a certain psychological anxiety. (P9)  There are complaints but, it's not the pain, it's what is behind it'. (P15) |

| **From the clinical examination to a diagnostic and treatment synthesis of a chronic idiopathic MSP** | | |
| --- | --- | --- |
| **Clinical examination** | **Absence of relevant objective elements toward a diagnosis** | It's the absence of any objective sign, and then I always try to trap them by distracting their attention by talking and then mobilizing the joints or limb segments that are said to be very painful. (P6)  A perfect growth curve is nonetheless one factor to make you say to yourself, it's probably not organic. It's major. (P4) |
|  | **Existence of sleep disorders without nocturnal**  **pain** | There is not really any nocturnal pain and nonetheless they are exhausted. (P7)  Generally, pain sleeps at night. (P15) |
|  | **Constellation of associated functional symptoms** | There are too many things at the same time to let me find a common point. (P1)  A constellation of signs, many too many signs.  (P12)  They all have a few, but when you ask for a little more detail, they are unable to provide them. (P21) |
| ***The elements of psychological semiology*** | **Relational difficulties with peers** | They are more mature than their peers, there's a kind of discrepancy. (P5)  Some are going to isolate themselves and then others are more with the adult world and less with their peers.(P5)  They are very often intelligent children, extremely good students, extremely nice. I am nonetheless struck by this profile, very well-behaved, very good students, sometimes a little obsessive, very applied. (P17) |
|  | **School absenteeism** | A lot of school absenteeism and no findings, it's a little suspicious. (P4) |
|  | **Performance anxiety** | He can say himself that he's very anxious, that he feels a lot of pressure. (P10) |
|  | **Catastrophizing** | There are also those who have nothing but who do not manage to be reassured with normal results and so you do more tests. What is important is to pinpoint this catastrophizing. (P19) |
|  | **A predominantly psychological dimension** | We see that there was in any case a psychological problem because there was no organic pathology. (P5)  When we have a doubt and besides, psychologically, the psychologist or whoever [tells us]: 'ah, look at the family: the father is in prison for molesting all of the siblings … ' It's perhaps not a side effect, but it's an expression of unhappiness… So it's sure that that's going to play an important role in the case, more or less. (P13) |
| ***The diagnostic procedure* and treatment tests** | **Clinical monitoring** | I see them again before 6 months is up, to see how things are evolving. (P7)  The course will make the diagnosis. (P7)  It took me more than six months to convince them, it's often a long process. (P9) |
|  | **Difficulties in using the diagnosis of fibromyalgia** | There's the problem of in-between, which is, which is really crucial, even in adults, it's the same thing, with patients who had real rheumatoid arthritis and fibromyalgia on top of that (P22).  I find that very complicated, this pain syndrome, to know what it is: there's an authentic pain that might be a moral pain that's explained by a physical pain perhaps. What is adult fibromyalgia? Finally I think that there is a lot that we don't know (P2).  Fibromyalgia, we attribute everything to it. I think it's a real disease, but it's not everything, and I think that's the problem (P15). |
|  | **Test treatment** | It's the ones with spondylarthritis that are a problem. So, if we're not sure, then the test for inflammation can be interesting in the sense if it works and shows spondylarthritis, all the better. (P13)  Sometimes, we are so stuck that we try biological therapies, anti-TNF trials, tests. It's rare, but true that sometimes we are nonetheless very stuck, the pain persists, when in any case it's enthesitis sites, we say we are going to try, and then we stop really fast. (P4) |
